# PI3K activation is associated with intracellular sodium/iodide symporter protein expression in breast cancer

**DOI:** 10.1186/1471-2407-7-137

**Published:** 2007-07-25

**Authors:** Katherine AB Knostman, James A McCubrey, Carl D Morrison, Zhaoxia Zhang, Charles C Capen, Sissy M Jhiang

**Affiliations:** 1 Department of Veterinary Biosciences, The Ohio State University, Columbus, Ohio 43210, USA; 2 Department of Microbiology and Immunology, Brody School of Medicine at East Carolina University, Greenville, North Carolina 27858, USA; 3 Department of Pathology, The Ohio State University, Columbus, Ohio 43210, USA; 4 Ohio State Biochemistry Program, The Ohio State University, Columbus, Ohio 43210, USA; 5 Department of Physiology and Cell Biology, The Ohio State University, Columbus, Ohio 43210, USA

## Abstract

**Background:**

The sodium/iodide symporter (NIS) is a membrane glycoprotein mediating active iodide uptake in the thyroid gland and is the molecular basis for radioiodide imaging and therapeutic ablation of thyroid carcinomas. NIS is expressed in the lactating mammary gland and in many human breast tumors, raising interest in similar use for diagnosis and treatment. However, few human breast tumors have clinically evident iodide uptake ability. We previously identified PI3K signaling as important in NIS upregulation in transgenic mouse models of breast cancer, and the PI3K pathway is commonly activated in human breast cancer.

**Methods:**

NIS expression, subcellular localization, and function were analyzed in MCF-7 human breast cancer cells and MCF-7 cells stably or transiently expressing PI3K p110alpha subunit using Western blot of whole cell lysate, cell surface biotinylation Western blot and immunofluorescence, and radioiodide uptake assay, respectively. NIS localization was determined in a human breast cancer tissue microarray using immunohistochemical staining (IHC) and was correlated with pre-existing pAkt IHC data. Statistical analysis consisted of Student's t-test (in vitro studies) or Fisher's Exact Test (in vivo correlational studies).

**Results:**

In this study, we demonstrate that PI3K activation in MCF-7 human mammary carcinoma cells leads to expression of underglycosylated NIS lacking cell surface trafficking necessary for iodide uptake ability. PI3K activation also appears to interfere with cell surface trafficking of exogenous NIS as well as *all-trans *retinoic acid-induced endogenous NIS. A correlation between NIS expression and upregulation of PI3K signaling was found in a human breast cancer tissue microarray.

**Conclusion:**

Thus, the PI3K pathway likely plays a major role in the discordance between NIS expression and iodide uptake in breast cancer patients. Further study is warranted to realize the application of NIS-mediated radioiodide ablation in breast cancer.

## Background

Breast cancer is one of the most common cancers in women in North America, where incidence is highest in the world [1]. In the United States, more than 200,000 newly diagnosed cases were estimated in the year 2005. While treatment with surgery, chemotherapy, hormonal and radiation therapy, and antibodies targeting the Her-2/neu growth promoting protein have slowed disease progression or resulted in remission in many patients, metastatic disease still causes death in the majority of affected patients within 5 years of diagnosis. Thus, continued discovery of novel methods of detecting and treating residual and metastatic breast cancer are of extremely high importance.

The sodium/iodide symporter (NIS) is a plasma membrane glycoprotein serving as the molecular basis for iodide accumulation in the thyroid gland, where it mediates active uptake of iodide from the bloodstream for incorporation into thyroid hormones [2]. Radioiodine is routinely administered to patients for the diagnosis and treatment of thyroid carcinomas, resulting in a 10-year survival rate of greater than 90% [3,4].

NIS is also expressed in the lactating mammary gland, where it facilitates uptake of iodide from the maternal bloodstream into milk for utilization by the developing neonatal thyroid gland [5,6]. While NIS has been detected in approximately 80% of human breast cancers using immunohistochemical staining [7], few tumors have clinically evident iodide uptake ability. In one study, although 24 out of 25 breast cancer patients had detectable NIS mRNA expression in their primary tumors, positive radionuclide uptake was only noted in four of the tumors (17%) and was correlated with NIS mRNA expression level [8]. A second study demonstrated that only 25% (2/8) of breast cancer metastases expressing NIS protein had clinically evident radionuclide uptake [9]. NIS-mediated radionuclide imaging and therapy has exciting potential as a safe and effective method of breast cancer diagnosis and treatment, and delineating factors underlying the discordance between NIS expression and function is critical in moving this modality into the practical realm. To achieve this aim, mechanisms of NIS regulation in breast cancer must be uncovered.

We previously found that phosphatidylinositol-3 kinase (PI3K) and cAMP signaling pathways were associated with NIS expression in transgenic mouse models of breast cancer [10]. However, cAMP alone was not sufficient to induce NIS protein expression in cultured MCF-7 human mammary carcinoma cells. Our current study was to evaluate the role of PI3K signaling in NIS expression, trafficking and iodide uptake ability in MCF-7 cells and in human breast tumors, as activating mutations of the PI3K p110α subunit are frequent in human breast cancers [11] and cell lines [12]. We found that stable or transient activation of PI3K p110α in MCF-7 cells increases underglycosylated NIS protein levels with impairment of NIS cell surface trafficking, which is required for iodide uptake activity. PI3K activation also interferes with cell surface trafficking and function of *all-trans *retinoic acid and hydrocortisone (tRAH) induced endogenous NIS as well as transiently expressed exogenous Flag-tagged NIS. Finally, an association was found between PI3K activation and NIS expression in human breast tumor tissues.

## Methods

### Cell culture

MCF-7 cells were maintained in medium consisting of equal parts phenol red-containing DMEM and Ham's F12, 10% fetal bovine serum (FBS) and 1% penicillin/streptomycin (Invitrogen, Carlsbad, CA). Two MCF-7 stable clones expressing PI3K p110α^CAAX ^were generated in the laboratory of Dr. James McCubrey at East Carolina University [13] and maintained in 89% RPMI (Invitrogen, Carlsbad, CA), 10% FBS, and 1% penicillin/streptomycin. In experiments requiring treatment with 1 μM *trans*-retinoic acid and 0.1 μM hydrocortisone (tRAH; Sigma, St. Louis, MO), both experimental and control groups of MCF-7 and MCF-7/PI3K p110α^CAAX ^cells were transferred to medium composed of equal parts DMEM and Ham's F12, 5% charcoal-stripped FBS and 1% penicillin/streptomycin.

### DNA constructs

PI3K p110α^CAAX ^was cloned into the BamHI site in the pcDNA3 vector. Flag-tagged human NIS open reading frame was cloned into the pcDNA3 vector as described in Zhang *et al*. [14]. Fugene6 reagent (Roche, Alameda, CA) was used for transient transfection according to the manufacturer's protocol. Co-transfection of a GFP expression plasmid was used to assess transfection efficiency.

### Cell surface biotinylation

Cell surface NIS expression was determined as described in Vadysirisack *et al*. [15]. Cells were surface biotinylated and lysed, followed by avidin pull-down using 5 mg of total cell lysate. Pulled-down biotinylated surface protein derived from MCF-7/PI3K p110α^CAAX ^cells and MCF-7 cells treated with tRAH for 48 hours [16] was used for Western Blot as described below.

### Western blot analysis

Western blot was performed as described in Jhiang *et al*. [17] using 100 μg of total cell lysates or membrane-enriched fraction (after removal of nucleus and cytosol) for total NIS, β-actin or Na^+^K^+^ATPase detection, or the pulled-down biotinylated surface protein, as described above, for surface NIS and Na^+^K^+^ATPase detection. Akt and pAkt Ser473 levels were assessed using 100 μg of whole cell lysate. Experiments utilized anti-hNIS antibody (#331, 1:1000), anti-actin antibody (Santa Cruz Biotechnology, Santa Cruz, CA; 1:200), anti-Akt and anti-pAkt Ser473 antibody (Cell Signaling Technology, Danvers, MA; 1:250) or anti-Na^+^K^+^ATPase antibody (Santa Cruz Biotechnology, Santa Cruz, CA; 1:1000). Secondary antibodies were anti-rabbit IgG conjugated to horseradish peroxidase for NIS, actin, Akt and pAkt Western blot (GE Healthcare Bio-Sciences Corp, Piscataway, NJ; 1:4000) or anti-mouse IgG conjugated to horseradish peroxidase for Na^+^/K^+^ATPase Western blot (Cell Signaling Technology, Danvers, MA; 1:4000). NIS antibody specificity was verified by PNGase F deglycosylation of NIS protein to a sole 50 kDa band, the molecular weight of underglycosylated NIS. Densitometry was performed by scanning with the Scion Image program (Scion Corp., Frederick, Md.).

### Immunofluorescence

NIS and Flag immunofluorescence were performed essentially as described in Marsee *et al*. [18]. Briefly parental MCF-7 cells and MCF-7/PI3K p110α^CAAX ^stable clones were seeded in 4-well chamber slides. Forty-eight hours after Flag-hNIS or empty vector transfection or tRAH treatment (when applicable), cells were fixed using 1% paraformaldehyde in PBS and labeled with anti-NIS antibody (#331; 1:1000) or anti-Flag M2 monoclonal antibody (Sigma, St. Louis, MO; 1:750), followed by incubation with Cy^TM3^-conjugated affinipure F(ab')2 fragment donkey anti-rabbit IgG (1:1400) for NIS immunofluorescence or anti-mouse IgG (Jackson ImmunoResearch Laboratories, West Grove, PA; 1:500) for Flag immunofluorescence. DAPI nuclear stain (Invitrogen, Carlsbad, CA) was applied before mounting and coverslipping. Secondary antibody only negative controls were included in the experiments. Images were obtained at 63× magnification using a Leica TCS SP2 AOBS Confocal Laser Scanning Microscope in The Ohio State University College of Veterinary Medicine.

### Radioactive iodide uptake assay

RAIU was performed as described in La Perle *et al*. [19] using MCF-7 or MCF-7/PI3K p110α^CAAX ^cells after forty-eight hours of tRAH treatment, Flag-hNIS or activated PI3K p110α^CAAX ^transient transfection (when applicable). In experiments involving transfection of MCF-7 cells and subsequent treatment with tRAH, the transfection time was set at 6 hours, followed by medium change and addition of tRAH for 42 hours. After incubation with ^125^I, washing and cell lysis, the supernatant was counted in a γ-radiation counter. Values were recorded in counts per minute (cpm) per microgram of DNA. Assays were performed in triplicate.

### Immunohistochemistry

Immunohistochemical staining technique was performed as described in Knostman *et al *[10] with the following modifications. A paraffin-embedded formalin-fixed human breast cancer tissue microarray consisted of 2 mm punches from 50 patient samples sectioned to 4 μm thickness and affixed to glass slides. Slides were incubated with rabbit polyclonal anti-human NIS primary antibody (#331; 1:500) followed by HRP-conjugated goat anti-rabbit IgG secondary antibody (Bio-Rad, Hercules, CA; 1:250) and chromogen DAB detection (Dako, Carpinteria, CA). Immunostaining for pAkt was performed in The Ohio State University Department of Pathology using mouse monoclonal anti-pAkt^Ser473 ^antibody (Cell Signaling Technology, Danvers, MA; 1:100). Thirty-three of the 36 tissues in the array had suitable integrity for interpretation of NIS positivity and pAkt expression and were included in the statistical analyses.

### Statistical analysis

Statistical analysis consisted of Student's t-test (in vitro studies) or Fisher's Exact Test (in vivo correlational studies) and was performed using GraphPad software. Only breast cancer tissue punches available for NIS, pAkt and PTEN immunohistochemical staining were used in statistical analyses. Additional comparison of NIS expression and estrogen receptor, progesterone receptor and Her-2/neu expression was performed, but no statistical relationship was evident.

## Results

**Figure 1:**
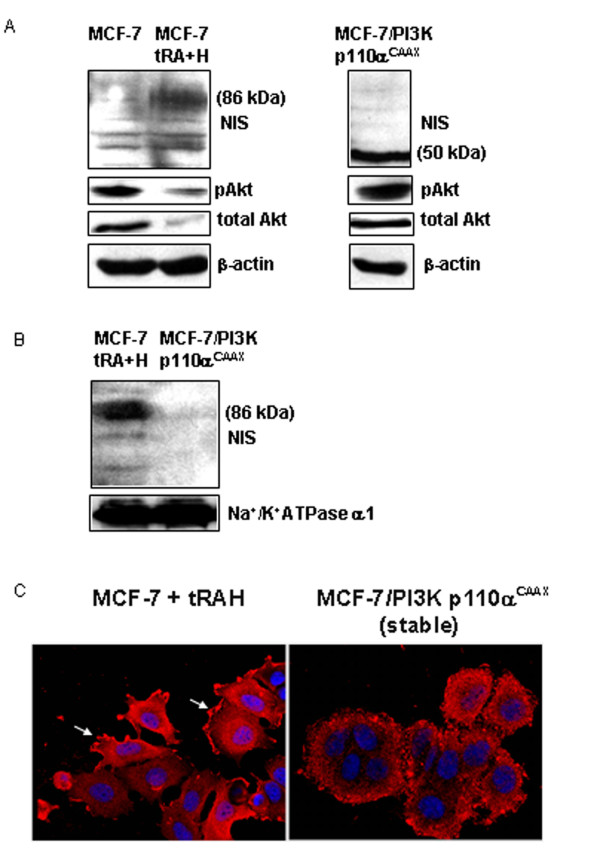
PI3K activation induces underglycosylated intracellular NIS protein expression in MCF-7 cells. (A) immunoblotting of membrane-enriched lysates from MCF-7 cells with and without tRAH treatment and MCF-7/PI3K p110α^CAAX ^stable clones using human NIS (hNIS) antibody. The specificity of NIS antibody was confirmed by conversion of the 86 kDa NIS band to a single 50 kDa band upon PNGase F deglycosylation (data not shown). Faint non-specific bands of 60 and 70 kDa are present. PI3K activation was assessed by total and phospho-Akt immunoblotting of total cell lysates. Actin was used as a loading control. (B) Immunoblotting of the surface protein fraction from MCF-7 cells treated with tRAH and MCF-7/PI3K p110α^CAAX ^stable clones using hNIS antibody. The surface fraction was isolated by cell surface biotinylation and avidin pull-down. Na^+^/K^+^ATPase was used as a loading control. (C) tRAH-treated MCF-7 cells and MCF-7/PI3K p110α^CAAX ^cells were labeled with hNIS antibody followed by Cy^TM3^-conjugated secondary antibody (red color) and DAPI nuclear stain (blue) for immunofluorescent microscopy. Parental MCF-7 cells and secondary antibody only controls were utilized, but are not shown. Magnification = 63×.

The importance of PI3K signaling in NIS regulation was studied using MCF-7 human mammary carcinoma cells, which are the only immortalized breast cancer cells with inducible NIS expression and function [20]. While NIS protein was not detectable in untreated MCF-7 cells, it was markedly induced by *all-trans *retinoic acid combined with hydrocortisone (tRAH) [16] as the glycosylated 86 kDa form (Figure 1A). In contrast, only the underglycosylated 50 kDa NIS form was present in MCF-7 cells stably expressing activated PI3K p110α^CAAX^. Cell surface NIS protein was detectable in MCF-7 cells treated with tRAH, but not in the MCF-7 cells stably expressing PI3K p110α^CAAX ^(Figure 1B). NIS immunofluorescence was performed to further assess the effect of PI3K activation on NIS subcellular localization in MCF-7 cells. While intense cell border NIS protein was evident in the tRAH-treated MCF-7 cells (arrows, left panel, Figure 1C) and sparsely located in the perinuclear area, it was not apparent in the MCF-7/PI3K p110α^CAAX ^cells, where NIS appeared dispersed throughout the intracellular compartment (right panel, Figure 1C).

**Figure 2:**
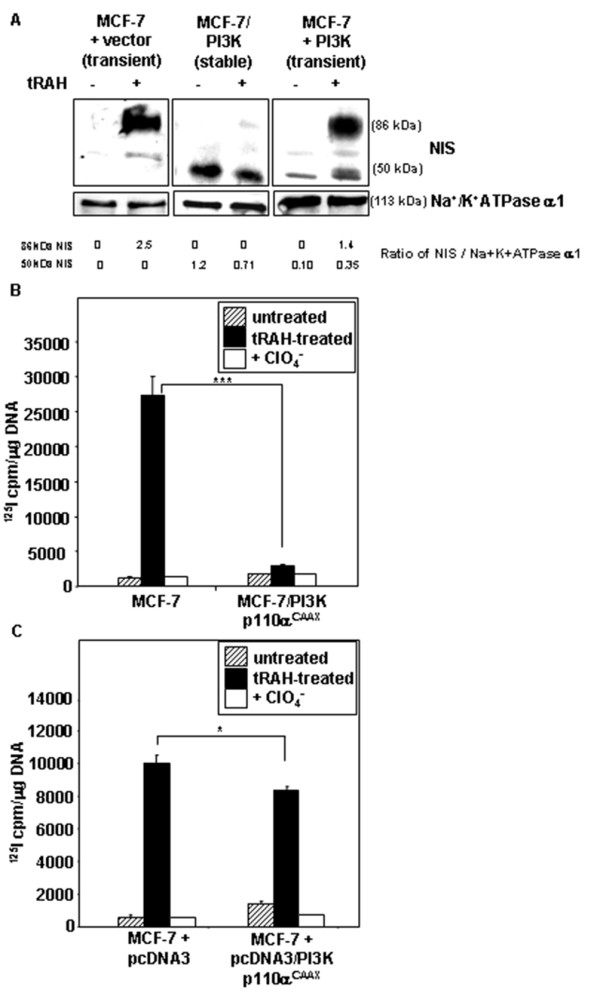
Stable or acute PI3K activation decreases tRAH-induced glycosylated NIS protein expression and NIS-mediated radioactive iodide uptake in MCF-7 cells. (A) 48 hours of tRAH treatment induces fully glycosylated NIS protein in MCF-7 cells transiently transfected with empty vector. Underglycosylated 50 kDa NIS is dominant in MCF-7/PI3K p110α^CAAX ^cells, which minimally respond to tRAH treatment. Transient expression of PI3K p110α^CAAX ^also induces underglycosylated NIS protein expression with a modest decrease in the tRAH-induced fully glycosylated NIS form. (B) tRAH treatment cannot induce NIS function in MCF-7/PI3K p110α^CAAX ^cells. A small increase in basal NIS-mediated radioactive iodide uptake is noted in MCF-7/PI3K p110α^CAAX ^stable clones versus parental MCF-7 cells. Cells were treated with tRAH for 48 hours followed by ^125^I uptake assay. Perchlorate (ClO_4_^-^) is a specific inhibitor of NIS function. ***p < .0001. (C) Acute expression of activated PI3K p110α decreases tRAH-induced NIS function in MCF-7 cells. A modest increase in basal NIS-mediated radioactive iodide uptake is present in MCF-7 cells transiently transfected with PI3K p110α^CAAX ^versus vector-only controls. Cells were transiently transfected with pcDNA3/PI3K p110α^CAAX ^or empty vector for 6 hours, and then treated with tRAH for 42 hours prior to ^125^I uptake assay. *p < .05.

PI3K activation also appears to interfere with the ability of tRAH to induce glycosylated NIS protein expression. As shown in Figure 2A, while abundant glycosylated NIS protein was induced by tRAH treatment in parental MCF-7 cells (lane 1 vs. lane 2), underglycosylated 50 kDa NIS was the dominant form in MCF-7/PI3K p110α^CAAX ^cells, which negligibly responded to tRAH treatment with glycosylated NIS protein (lane 3 vs. lane 4). Transient expression of PI3K p110α^CAAX ^also induced the underglycosylated 50 kDa NIS form (lane 1 vs. lane 5), and responsiveness to tRAH treatment with glycosylated NIS protein was decreased by approximately 45% (lane 2 vs. lane 6) even though the transfection efficiency was only 20–25%. Interestingly, tRAH treatment slightly decreased the overall level of NIS protein in MCF-7/PI3K p110α^CAAX ^cells.

MCF-7/PI3K p110α^CAAX ^cells initially had modest basal iodide uptake ability, as published in our previous study [10], but most of this function was lost over cell passage even though NIS protein remained detectable. Radioiodide uptake ability in our current study correlated well with fully glycosylated (surface) NIS protein levels. The stably transfected MCF-7/PI3K p110α^CAAX ^cells had minimally increased NIS-mediated radioiodide uptake over parental MCF-7 cells and did not significantly respond to tRAH treatment (Figure 2B). Transient transfection of activated PI3K p110α^CAAX ^in parental MCF-7 cells modestly increased NIS-mediated radioiodide uptake over empty vector-transfected controls but decreased tRAH-induced radioiodide uptake activity (Figure 2C).

**Figure 3:**
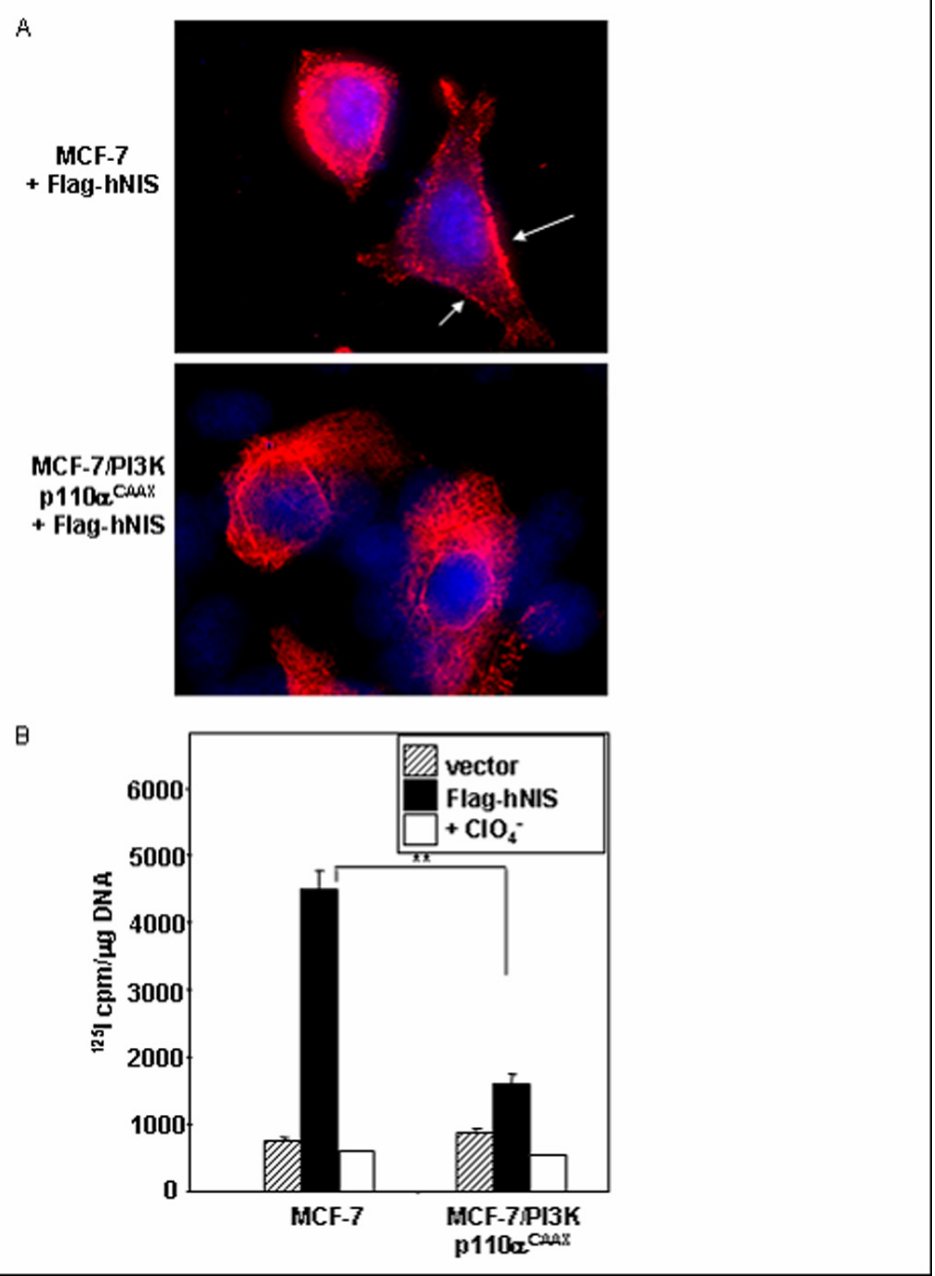
PI3K activation interferes with cell surface trafficking and radioiodide uptake conferred by exogenous Flag-tagged hNIS gene expression. (A) MCF-7 and MCF-7/PI3K p110α^CAAX ^cells were transiently transfected with pcDNA3/Flag-hNIS and subjected to Flag immunofluorescence 48 hours later. While Flag-hNIS is detectable on the cell surface in parental MCF-7 cells (arrows), it is retained intracellularly in MCF-7/PI3K p110α^CAAX ^cells. Magnification = 63×. (B) MCF-7 and MCF-7/PI3K p110α^CAAX ^cells were transiently transfected with pcDNA3/Flag-hNIS and subjected to ^125^I uptake assay 48 hours later. A nearly 4-fold increase in ^125^I uptake resulting from exogenous NIS expression is present in parental MCF-7 cells as compared to MCF-7/PI3K p110α^CAAX^. **p < .001.

A similar inhibitory effect of PI3K activation on NIS surface trafficking and function was observed upon forced expression of exogenous NIS. Immunofluorescence for exogenous Flag-tagged NIS protein demonstrated that Flag-NIS cell surface trafficking was largely inhibited in MCF-7/PI3K p110α^CAAX ^cells as compared to parental MCF-7 cells (Figure 3A). Additionally, radioiodide uptake activity conferred by transient transfection of exogenous Flag-NIS was decreased by 4-fold in MCF-7/PI3K p110α^CAAX ^cells versus parental MCF-7 cells (Figure 3B). Thus, cell surface trafficking of both endogenous and exogenous NIS were decreased by PI3K activation.

To determine whether PI3K upregulation plays a role in the discordance between the increase in NIS expression and the absence of radioiodide uptake activity in human breast tumors, we evaluated a human breast cancer tissue microarray for NIS expression and localization using immunohistochemistry. This array consisted of randomly selected breast tissues from patients at The Ohio State University Medical Center with a diagnosis of breast carcinoma during the years 1992–1994. Tumors were 51% ER-positive, 57% PR-positive, 53% Her-2/neu-positive, and 27% with loss of PTEN tumor suppressor, each of which had no statistical correlation with NIS expression. All tumor specimens were primary, although 29% of the patients also had local lymph node metastases. None had known distant metastases at the time of biopsy. Patients had an average age of 42.26 years and were 89% Caucasian and 11% African-American.

**Figure 4:**
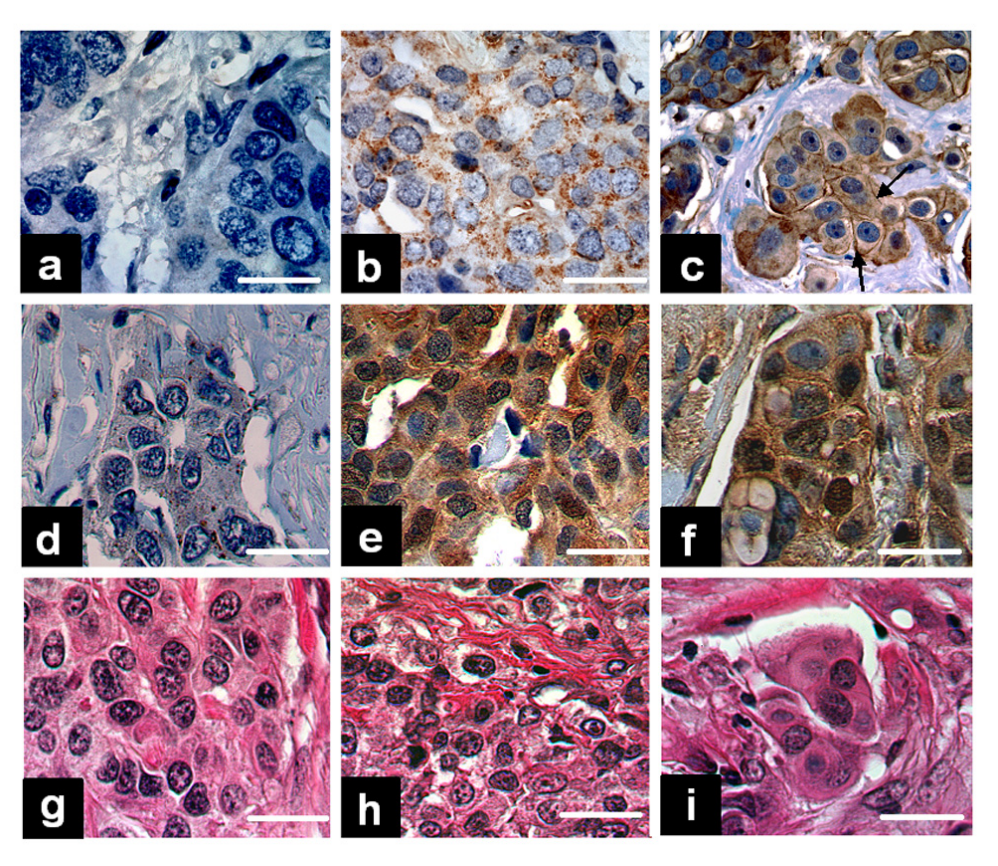
NIS is expressed in 80% of human breast tumors in a tissue microarray with primarily intracellular localization and positive correlation with pAkt expression. (A-C) NIS immunohistochemical staining in three representative breast cancer specimens. (A) NIS-negative breast tumor. (B) Breast tumor with intracellular NIS localization. (C) Breast tumor with both intracellular and plasma membrane-localized NIS protein (arrows). (D-F) Immunohistochemical staining for pAkt in the three breast tumors shown in A-C. (G-I) H & E staining demonstrating the histopathology of the three breast tumors shown in A-C. Bar = 20 μM.

We correlated NIS expression/localization with the presence of PI3K activation as indicated by phosphorylation and nuclear translocation of Akt, which is commonly used to estimate PI3K pathway activation. Similar to previously published data, only 20% of tumors were NIS-negative (Figure 4A). Overall, 58% of tumors had primarily intracellular NIS expression (Figure 4B), while 22% had some degree of plasma membrane NIS expression (Figure 4C). NIS-positive tumors were three times more likely than NIS-negative tumors to have PI3K activation as indicated by Akt phosphorylation (Table 1 and Figures 4A–C versus 4D–F). Interestingly, all tumors with loss of the PTEN (PI3K antagonist) and concomitant pAkt expression had marked intracellular NIS expression without notable cell surface NIS expression.

**Table 1:** Correlation of NIS expression and localization with Akt activation and localization

**Tumor classification**	**nuclear pAkt**		**any pAkt**		**any pAkt +loss of PTEN**	
	*n*	*%*	*n*	*%*	*n*	*%*
NIS-negative tumors	2/7	28	2/7	28*	0/7	0
NIS-positive tumors	16/26	62	19/26	73*	4/25	16
*NIS intracellular (only)*	12/19	63	14/19	74	4/18	22
*NIS plasma membrane (any)*	4/7	57	5/7	71	0/7	0

* p < .05

## Discussion

This study is the first to uncover a potential explanation for the discordance between NIS expression and iodide uptake ability in human breast cancers, a roadblock to widespread application of NIS-mediated radioiodide imaging and ablation of residual and metastatic tumors. We showed that both transient and chronic PI3K activation increase underglycosylated NIS protein levels but impair NIS cell surface trafficking, thus accounting for the absence of NIS-mediated radioiodide uptake activity. NIS expression and intracellular localization were also associated with pAkt expression in human breast cancer tissues. Our study suggests that the increased intracellular NIS protein in human breast cancer specimens [7] is likely a consequence of PI3K activation.

Many human breast cancers have PI3K activation as a consequence of Her-2/neu or Src oncogene expression [21], or by activating mutations of the PI3K p110α subunit [11], any of which could lead to NIS expression. In our breast cancer tissue microarray, PI3K activation might be explained by Her-2/neu expression in 48% of the pAkt-positive tumors or by loss of PTEN in 19% of pAkt-positive tumors. Activating point mutation E545K in the helical domain of PI3K p110α has been described in many cultured breast cancer cells, including MCF-7 [12]. In our study, both chronic and transient PI3K p110α overexpression resulted in 2–3 fold greater pAkt protein levels than found in parental MCF-7 cells.

The lack of NIS protein glycosylation in PI3K p110α^CAAX^-expressing MCF-7 cells is consistent with impairment of processing for NIS cell surface trafficking. Membrane glycoproteins are processed by glycosylation beginning in the endoplasmic reticulum and Golgi apparatus with transport using machinery such as the AP-1B adaptor complex and clathrin-coated vesicles and final transport to the cell surface by carrier vesicles [22]. While it is not currently known how PI3K activation interferes with NIS cell surface trafficking, it is known that one subtype of PI3K interacts with clathrin [23].

It has been shown that abolishing three glycosylation sites in human NIS resulted in a 50 kDa NIS protein with decreased NIS activity after exogenous delivery into COS cells [24]. The fact that this mutant still had 50% of wild-type iodide uptake ability indicates that some NIS protein was able to reach to the cell surface. However, it was unclear whether the decrease in radioiodide uptake was due to less efficient cell surface trafficking or to a decrease in NIS activity. We interpret the lack of NIS glycosylation in our MCF-7 model to be a consequence of protein retention at a pre-glycosylation stage rather than a cause of trafficking failure. However, we cannot exclude that a lack of glycosylation itself might impair trafficking and function.

In our current study, tRAH-responsive NIS induction was nearly abolished in MCF-7/PI3K p110α^CAAX ^cells and reduced in parental MCF-7 cells transiently transfected with PI3K p110α^CAAX^. The greater inhibition of NIS cell surface trafficking and function in PI3K-overexpressing stable clones vs. PI3K-transfected MCF-7 cells could be related to chronicity of PI3K activation, which is a likely scenario in human breast cancer. Additionally, transient transfection efficiency is only 25–30% in MCF-7 cells.

In summary, this study advances our knowledge of NIS regulation in breast cancer by demonstrating that PI3K signaling increases underglycosylated NIS protein levels without an increase in NIS-mediated radioiodide uptake activity due to impairment in NIS cell surface trafficking. Because many oncogenes in human breast cancer upregulate PI3K signaling, and PI3K somatic mutation was frequently detected in human breast cancers, this pathway could realistically account for the high intracellular NIS expression and lack of radioiodide uptake in most human breast cancers. The fact that PI3K signaling appears to have differential effects on NIS expression in the breast versus thyroid suggests that a strategy may be developed to selectively increase NIS expression in breast tissue. Further investigation into the mechanism(s) of impairment of NIS cell surface trafficking by PI3K will be critical in realizing the application of NIS-mediated radioiodide imaging and ablation in breast cancer.

## Abbreviations

NIS: Sodium/iodide symporter

RAIU: Radioactive iodide uptake assay

tRAH: all-trans retinoic acid and hydrocortisone

## Competing interests

The author(s) declare that they have no competing interests.

## Authors' contributions

KK carried out the molecular genetic studies and immunohistochemical staining and drafted the manuscript. JM created the MCF-7 cells stably expressing PI3K p110α^CAAX^, provided the PI3K p110α^CAAX ^DNA construct and contributed to the discussion section of the manuscript. CM created and provided the human breast cancer tissue microarray and assisted in interpretation of immunohistochemical staining results. ZZ created the Flag-tagged human NIS construct and assisted with experiments using the construct. CC and SJ conceived of the study and participated in its design and coordination. SJ provided the laboratory environment and support necessary for completion of the study. All authors read and approved the final manuscript.

## Pre-publication history

The pre-publication history for this paper can be accessed here:


